# Adropin Predicts Asymptomatic Heart Failure in Patients with Type 2 Diabetes Mellitus Independent of the Levels of Natriuretic Peptides

**DOI:** 10.3390/diagnostics14161728

**Published:** 2024-08-09

**Authors:** Tetiana A. Berezina, Oleksandr O. Berezin, Uta C. Hoppe, Michael Lichtenauer, Alexander E. Berezin

**Affiliations:** 1 Department of Internal Medicine and Nephrology, VitaCenter, 69000 Zaporozhye, Ukraine; talexberezina@gmail.com; 2 Luzerner Psychiatrie AG, 4915 St. Urban, Switzerland; lunik.mender@gmail.com; 3 Department of Internal Medicine II, Division of Cardiology, Paracelsus Medical University, 5020 Salzburg, Austria; u.hoppe@salk.at (U.C.H.); m.lichtenauer@salk.at (M.L.)

**Keywords:** adverse cardiac remodeling, heart failure with preserved ejection fraction, natriuretic peptides, adropin, circulating biomarkers

## Abstract

In patients with type 2 diabetes mellitus (T2DM), asymptomatic adverse cardiac remodeling plays a pivotal role in the development of heart failure (HF). Patients with T2DM often have low or near-normal levels of natriuretic peptides, including N-terminal brain natriuretic peptide (NT-proBNP), which have been inconclusive in predicting the transition from asymptomatic adverse cardiac remodeling to HF with preserved ejection fraction (HFpEF). The aim of this study was to elucidate the predictive ability of adropin for HFpEF depending on the circulating levels of NT-proBNP. We prospectively enrolled 561 T2DM patients (glycated hemoglobin < 6.9%) with echocardiographic evidence of structural cardiac abnormalities and left ventricular ejection fractions >50%. All patients underwent B-mode transthoracic echocardiographic and Doppler examinations. Circulating biomarkers, i.e., NT-proBNP and adropin, were assessed at baseline. All individuals were divided into two groups according to the presence of low levels (<125 pmol/mL; *n* = 162) or elevated levels (≥125 pmol/mL; *n* = 399) of NT-proBNP. Patients with known asymptomatic adverse cardiac remodeling and elevated NT-proBNP were classified as having asymptomatic HFpEF. A multivariate logistic regression showed that low serum levels of adropin (<3.5 ng/mL), its combination with any level of NT-proBNP, and use of SGLT2 inhibitors were independent predictors of HFpEF. However, low levels of adropin significantly increased the predictive ability of NT-proBNP for asymptomatic HFpEF in patients with T2DM, even though the concentrations of NT-proBNP were low, while adropin added discriminatory value to all concentrations of NT-proBNP. In conclusion, low levels of adropin significantly increase the predictive ability of NT-proBNP for asymptomatic HFpEF in patients with T2DM.

## 1. Introduction

Asymptomatic adverse cardiac remodeling is defined as structural (cardiac hypertrophy) or functional (local contractility dysfunction or diastolic abnormalities with a normal/near-normal ejection fraction) cardiac abnormalities in the absence of signs/symptoms of heart failure (HF), which are included in the definition of stage B HF [1]. The transition from asymptomatic adverse cardiac remodeling to symptomatic HF is associated with poor clinical outcomes and is mediated by multiple risk factors (aging, hypertension, chronic kidney disease, and arrhythmia) and causative cardiovascular (persistent ischemia and necrosis) and non-cardiovascular (obesity, diabetes, thyroid dysfunction, autoimmune conditions, cardiac amyloidosis and sarcoidosis, inflammation, polychemotherapy of malignancy, etc.) factors [2,3]. The new recommendations for HF from the recent American College of Cardiology (ACC)/American Heart Association (AHA)/Heart Failure Society of America (HFSA) guidelines and the European Society of Cardiology (ESC) Scientific Group clearly indicate that early diagnosis and treatment of adverse cardiac remodeling may prevent an unfavorable course of HF [4,5]. However, better classification of the echocardiographic phenotype (diastolic changes with or without structural remodeling), together with the detection of elevated levels of circulating cardiac biomarkers, such as brain natriuretic peptide (BNP)/N-terminal brain natriuretic peptide (NT-proBNP) and high-sensitivity cardiac troponin, is considered an effective approach to determine morbidity and mortality as well as the risk of hospitalization [6,7,8]. On the contrary, natriuretic peptides showed sufficient variability in their predictive values for the development and progression of HF in individuals of different ages with obesity and type 2 diabetes mellitus (T2DM), chronic kidney disease (CKD), and atrial fibrillation [9]. The concept of ‘biomechanical stress’, when NT-proBNP levels are elevated in an asymptomatic patient with adverse cardiac remodeling, has limited predictive value for HF with preserved ejection fraction (HFpEF), whereas it was found to be sufficiently powerful for HF with reduced ejection fraction (HFrEF). It remains unclear whether this concept has similar predictive value for HFpEF in patients with low NT-proBNP levels due to the presence of metabolic HF risk factors such as T2DM and obesity [10,11]. In this context, the discovery of a novel biomarker approach to detect the risk of adverse cardiac remodeling—the transition to HF—in individuals with low NT-proBNP appears promising.

Adropin is a multifunctional hepatokine that is primarily produced by hepatocytes and the hypothalamus and regulates glucose homeostasis, free fatty acid oxidation, and insulin resistance in skeletal muscle and the myocardium, as well as hepatosteatosis, independent of food intake, body weight, and energy expenditure [12,13]. Adropin down-regulates the phosphorylation of pyruvate dehydrogenase kinase 4 and the inhibitory phosphorylation of c-Jun N-terminal kinase, thereby attenuating the functional and structural integrity of mitochondria [14]. The central nervous system actions of adropin include suppression of water intake and regulation of blood pressure through the orphan G protein-coupled receptor (GPR), which is a potential adropin receptor [15]. Interestingly, adropin-mediated GPR19 signaling appears to be involved in the p44/42 mitogen-activated protein kinase (MAPK) pathway, which regulates mitochondrial glucose utilization pathways and suppresses oxidative stress in cardiac cells [16]. Furthermore, in in vitro studies, adropin suppressed the hydrogen peroxide-induced endothelial-to-mesenchymal transition and the progression of atherosclerosis by decreasing transforming growth factor (TGF)-β1 and TGF-β2 mRNA and protein expression and suppressing phosphorylation of the downstream signaling protein Smad2/3 [17,18]. Finally, adropin plays a key role in maintaining blood–brain barrier integrity, vasodilation, and blood pressure control via an endothelial nitric oxide synthase (eNOS)-dependent mechanism [19,20].

In clinical settings, low levels of adropin have been found in individuals with arterial hypertension, obesity, T2DM, metabolic syndrome, coronary artery disease, atherosclerosis, stroke, acute coronary syndrome, chronic kidney disease, and all phenotypes of symptomatic HF [21,22,23,24,25,26,27]. Adropin appears to exert a protective effect on the endothelium and cardiac myocytes and has been suggested as a biomarker of endothelial dysfunction, adverse cardiac remodeling, renal failure, and HF [28,29,30]. However, there is no clear evidence that adropin can provide additional predictive information about HFpEF in asymptomatic individuals with low levels of natriuretic peptides. The aim of this study was to clarify the predictive role of adropin for HFpEF as a function of circulating NT-proBNP levels.

## 2. Materials and Methods

### 2.1. Patient Population and Study Design

**Figure 1:**
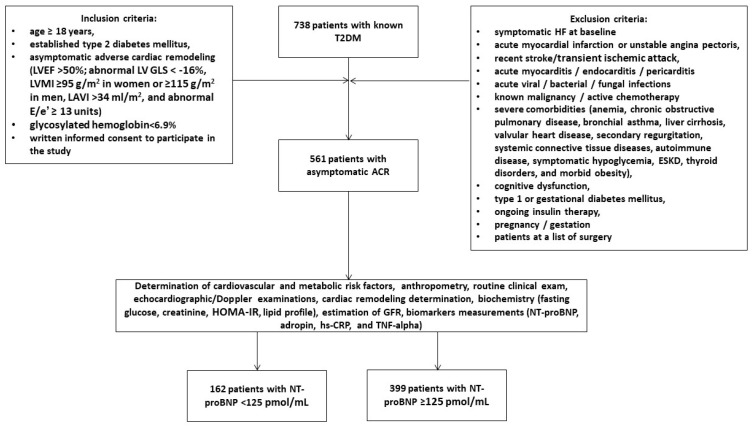
Flow chart with study design. Abbreviations: ACR, adverse cardiac remodeling; E/e′, ratio of early transmitral diastolic filling velocity/early mitral annular velocity; eGFR, estimated glomerular filtration rate; hs-CRP, high-sensitivity C-reactive peptide; GLS, global longitudinal strain; LV, left ventricular; LAVI, left atrial volume index; LVEF, left ventricular ejection fraction; LVMI, LV mass index; NT-proBNP, N-terminal brain pro-peptide; T2DM, type 2 diabetes mellitus; TNF, tumor necrosis factor.

A total of 738 patients with T2DM were identified through a local database related to the “Vita Center” (Zaporozhye, Ukraine). The following inclusion criteria were used: individuals of both genders with ages ≥ 18 years, established T2DM, asymptomatic adverse cardiac remodeling, glycosylated hemoglobin (HbA1c) < 6.9%, and informed consent to participate in this study. We prospectively enrolled 561 T2DM patients who had evidence of structural cardiac abnormalities (Figure 1). We used the following criteria to classify the structural cardiac abnormalities: left ventricular ejection fraction [LVEF] > 50%, abnormal left ventricular (LV) global longitudinal strain (GLS) < −16%, LV hypertrophy (LVH), left atrial volume index (LAVI) > 34 mL/m^2^, and an abnormal ratio of early transmitral diastolic filling velocity/early mitral annular velocity (E/e′) ≥ 13 units [5]. LVH was detected as an increased LV mass index (LVMI) ≥ 95 g/m^2^ in women or ≥115 g/m^2^ in men [31].

The exclusion criteria were symptomatic acute or chronic HF, acute coronary syndrome/myocardial infarction or unstable angina pectoris, recent strokes/transient ischemic attacks, acute myocarditis/endocarditis/pericarditis, known malignancies and/or chemotherapy, acute viral/bacterial/fungal infections, severe co-morbidities (anemia, chronic obstructive pulmonary disease, bronchial asthma, liver cirrhosis, known inherited and acquired valvular heart defects, symptomatic severe hypoglycemia, morbid obesity, systemic connective tissue diseases, end-stage renal disease, autoimmune disease, cognitive dysfunction, and thyroid disorders), pregnancy/gestation, type 1 or gestational diabetes mellitus, and current therapy with insulin.

All individuals were divided into two groups depending on the presence of NT-proBNP < 125 pmol/mL (*n* = 162) or ≥125 pmol/mL (*n* = 399). Patients with adverse cardiac remodeling and elevated NT-proBNP were classified as having asymptomatic HFpEF.

### 2.2. Collection of Relevant Medical Data and Background Information

Demographic and anthropomorphic data, basic clinical characteristics, and comorbidities were collected at baseline.

### 2.3. Echocardiographic Examination

All patients underwent echocardiographic and Doppler examinations by two blinded, highly experienced echocardiographers according to the guidelines of the American Society of Echocardiography [31]. Standard apical two- and four-chamber views were used to obtain baseline data, and the examinations were repeated with a 52-week observation interval. This was performed using a HealtheCare Vivid E95 scanner (General Electric Company, Horton, Norway). Conventional hemodynamic parameters included the left ventricular ejection fraction (LVEF) (obtained by Simpson’s method), the left ventricular end-diastolic (LVEDV) and end-systolic (LVESV) volumes, the left atrial volume index (LAVI), early diastolic blood filling (E), and the mean longitudinal strain ratio (e′). The estimated E/e′ ratio was expressed as the ratio of the E-wave velocity to the averaged medial and lateral e′ velocity. The LV GLS was obtained by 2D speckle-tracking image analysis after acquisition of high-quality echocardiographic data during at least three cardiac cycles. The data were stored in the DICOM format for subsequent analysis.

### 2.4. Blood Sampling and Biomarker Analysis

Venous blood samples (3–5 mL) were collected from fasting patients in Vacutainer tubes at three time points: baseline, 26 weeks, and the end of the study. Pooled samples were centrifuged (3000 rpm for 30 min). Sera were collected and immediately snap-frozen and stored at −70 °C until analysis. All routine biochemical tests were performed using standard biochemical techniques on a Roche P800 analyzer (Basel, Switzerland).

The concentrations of NT-proBNP, adropin, tumor necrosis factor (TNF)-alpha, and high-sensitivity C-reactive protein (hs-CRP) were determined in the serum samples using commercial enzyme-linked immunosorbent assay (ELISA) kits from Elabscience (Houston, TX, USA). The intra- and inter-assay coefficients of variation were less than 10% for all kits.

### 2.5. Glomerular Filtration Rate and Insulin Resistance Determination

The conventional CKD-EPI formula was used to estimate the glomerular filtration rate (eGFR) [32]. The Homeostatic Assessment Model of Insulin Resistance (HOMA-IR) was used to assess insulin resistance [33].

### 2.6. Statistics

The Kolmogorov–Smirnov test was used to assess the normality of the data, while the Levene test was used to assess the homogeneity of the data. Continuous variables were expressed either as means (Ms) and standard deviations (SDs) or as medians (Mes) and 25–75% interquartile range (IQRs), depending on the distributions of the data. Categorical variables were expressed as proportions and percentages of the totals. Chi-square, Mann–Whitney U, and Kruskal–Wallis tests were used to determine the statistical significance of differences in variance according to the distributions. Spearman’s correlation coefficients were calculated to determine the correlations between the variables. Plausible predictors of HFpEF were identified using univariate and multivariate logistic regressions. The odds ratio (OR), 95% confidence interval (CI), and Harrell’s concordance index (c-index) were calculated for each predictor. The reliability of the predictive models was determined by receiver operating curve (ROC) analysis, which included calculating the area under the curve (AUC). The incremental predictive ability of the models was compared to a binary prediction method based on the estimation of integrated discrimination indices (IDIs) and net reclassification improvement (NRI). A two-tailed *p*-value less than 0.05 was considered statistically significant. Variables were tested using SPSS v. 23 (IBM, Armonk, New York, NY, USA) and GraphPad Prism v. 9 (GraphPad Software, San Diego, CA, USA).

## 3. Results

The patients enrolled in this study had a mean age of 58 years, and 51.7% were male (Table 1). They had an average diabetes duration of 11.20 ± 6.75 years, a mean body mass index (BMI) of 26.03 ± 5.20 kg/m^2^, a mean waist circumference of 96.60 ± 3.60 cm, and an average waist-to-hip ratio of 0.88 ± 0.10 units. The patients had a comorbidity profile that included dyslipidemia (77.9%), hypertension (74.0%), stable coronary artery disease (30.9%), atrial fibrillation (16.9%), smoking (41.0%), abdominal obesity (38.9%), left ventricular hypertrophy (90.7%), and chronic kidney disease stages 1–3 (29.6%). All patients were hemodynamically stable and had a mean LVEF of 54%, a mean LAVI of 42 mL/m^2^ (35–50 mL/m2), a mean E/e′ of 16 ± 5, and a mean GLS of −14.8% (−12.7; −15.7%). They also had a mean HOMA-IR score of 7.12 ± 2.7, a mean HbA1c of 6.45 ± 0.18%, a mean fasting plasma glucose of 6.42 ± 1.9 mmol/L, a mean eGFR of 79 mL/min/1.73 m^2^, a mean plasma creatinine of 100.9 ± 20.1 µmol/L, and a mean plasma serum uric acid of 321 ± 115 mcmol/L.

The lipid profile was consistent with dyslipidemia. The levels of the inflammatory cytokines hs-CRP and TNF-alpha were 4.39 mg/L (1.98–6.80 mg/L) and 2.70 pg/mL (1.90–3.62 pg/mL), respectively. The plasma concentrations of NT-proBNP and adropin were 147 pmol/mL (65–231 pmol/mL) and 3.50 ng/mL (2.05–4.67 ng/mL), respectively. All individuals received optimal therapy, including diet and drugs, according to their glycemic status and comorbidity profile. We found no differences between the groups of patients in terms of sex, duration of T2DM, anthropometric characteristics, frequency of dyslipidemia, hypertension, abdominal obesity, CKD, systolic and diastolic blood pressure, LV cavity dimensions, LVEF, LAVI, E/e′, GLS, HOMA-IR score, fasting glucose, HbA1c, creatinine, lipids, hs-CRP, TNA-alpha, or medications (excluding antiplatelet agents/anticoagulants and SGLT2 inhibitors). However, patients with low NT-proBNP were older; less likely to have stable coronary artery disease (CAD), LVH, and atrial fibrillation; and had significantly lower eGFR and serum uric acid levels and higher adropin levels than those with elevated NT-proBNP. In addition, patients with low NT-proBNP were less likely to be treated with ACE inhibitors, angiotensin II receptor blockers, and sodium–glucose cotransporter-2 (SGLT2) inhibitors than those with elevated NT-proBNP. On the contrary, they were more likely to receive antiplatelet agents and anticoagulants than those with elevated NT-proBNP.

### 3.1. Spearman’s Correlations between the Levels of Biomarkers and Other Parameters

The NT-proBNP levels were positively associated with atrial fibrillation (r = 0.36, *p* = 0.001), E/e′ (r = 0.34, *p* = 0.001), the LAVI (r = 0.31, *p* = 0.001), LVH (r = 0.27, *p* = 0.044), and age (r = 0.22, *p* = 0.048) and inversely correlated with GLS (r = −0.35, *p* = 0.001) and the eGFR (r = −0.32, *p* = 0.001). The adropin levels were positively associated with GLS (r = 0.38, *p* = 0.001) and negatively correlated with the LAVI (r = −0.32, *p* = 0.001), fasting plasma glucose (r = −0.30, *p* = 0.001), HOMA-IR scores (r = −0.27, *p* = 0.042), age (r = −0.26, *p* = 0.001), BMI (r = −0.24, *p* = 0.001), and HbA1c (r = −0.23, *p* = 0.046). The adropin levels were not significantly associated with the presence of CAD, hypertension, or CKD or the duration of T2DM, whereas there was a positive association between adropin and atrial fibrillation. We did not find significant associations between inflammatory biomarkers and NT-proBNP, adropin, or components of the lipid profile.

### 3.2. Predictors of Asymptomatic HFpEF: Univariate and Multivariate Logistic Regression Analyses

For further regression analysis, we used cut-off points of 125 pmol/mL and 3.5 ng/mL for NT-proBNP and adropin, respectively. The cut-off point for adropin identified concentrations above and below the median (3.5 ng/mL). However, we included the following variables in the regression constructed from the combined biomarkers, i.e., NT-proBNP and adropin: low (<125 pmol/mL) and elevated (≥125 pmol/mL) concentrations of NT-proBNP and low (<3.5 ng/mL) and elevated (≥3.5 ng/mL) concentrations of adropin.

In a univariate logistic regression, low serum levels of NT-proBNP did not predict HFpEF, whereas low adropin levels, atrial fibrillation, LVH, and the use of SGLT2 inhibitors were found to be predictors of HFpEF (Table 2). In line with this, low levels of adropin increased the discriminatory power of NT-proBNP independent of its level. A multivariate logistic regression showed that low adropin, its combination with any level of NT-proBNP, and the use of SGLT2 inhibitors remained independent predictors of HFpEF.

### 3.3. Comparison of the Predictive Models

We compared the predictive models for HFpEF and found that Model 1 (low levels of adropin) was not significantly better than Model 3 (elevated NT-proBNP + low adropin), whereas Model 2 (low NT-proBNP + low adropin) had the same discriminative potency as the reference value (Table 3). Model 4 did not outperform Model 1 in terms of its predictive value.

## 4. Discussion

This study first showed that low levels of adropin significantly increased the predictive ability of NT-proBNP for asymptomatic HFpEF in patients with T2DM, even when the NT-proBNP levels were low, while adropin added discriminatory power at all NT-proBNP levels. Thus, adropin appears to overcome the inadequate predictive value of reduced or subnormal natriuretic peptide concentrations for the conversion from ACR to HFpEF in patients with metabolic risk factors, including diabetes mellitus and obesity, and it predicts asymptomatic HFpEF.

Previous studies have shown that elevated plasma levels of natriuretic peptides have a strong negative diagnostic value for HF, while their discriminatory power was found to be higher for HFrEF compared to HFpEF [34,35]. At the same time, NT-proBNP remains a prognostic biomarker for adverse cardiac remodeling and any phenotype of HF [36,37]. Although there is an inverse association between NT-proBNP levels and T2DM in individuals without cardiovascular disease, individuals with CVD, including LVH and adverse cardiac remodeling, often have near-normal levels of NT-proBNP [38]. However, atrial fibrillation is a strong factor associated with both T2DM and HFpEF/HFrEF and contributes to increases in natriuretic peptides independent of the presence of metabolic abnormalities [39]. Indeed, we found that NT-proBNP levels >125 pmol/L were common in individuals with T2DM and atrial fibrillation. However, regardless of any differences in age, body composition, and the comorbidity profile (CAD, LVH, atrial fibrillation, and chronic kidney disease), elevated levels of NT-proBNP (>3000 pg/mL) retained their ability to predict all-cause mortality and cardiovascular death in patients with both HFpEF and HFrEF [40,41]. However, near-normal levels of NT-proBNP associated with obesity, metabolic syndrome, and T2DM appear to have limited utility in predicting adverse cardiac remodeling, the transition to HFpEF, and the incidence of HFpEF [42]. Although a higher body mass index was found to be the strongest predictor of unexpectedly low natriuretic peptides in HF patients, in our study we found no difference in BMI between patients with adverse cardiac remodeling and HFpEF. We suggest that a possible cause for the existence of natriuretic peptide deficiency is dysregulation of their metabolism, including clearance and expression of natriuretic peptide receptors in the liver, kidneys, skeletal muscle, and adipose tissue, which may be enhanced by adipokine and hepatokine release, concomitant inflammation-induced lipolysis, and energy expenditure. [43].

Adropin—a multifunctional hepatokine—was previously shown to have organ-protective properties and has been detected at low levels in T2DM and HF [44,45,46]. Furthermore, a trend towards increased adropin was associated with beneficial changes in cardiac function and attenuation of renal outcomes in HF patients with T2DM [29,45,46]. However, it remained questionable whether adropin could add specific predictive information to NT-proBNP, thereby improving its discriminatory power in asymptomatic HFpEF over a wide range of circulating levels. The results of our study showed the utility of the predictive model constructed using adropin and NT-proBNP for asymptomatic HFpEF in diabetic patients, even when the NT-proBNP levels were near-normal or slightly elevated. These findings offer a new perspective for understanding the roles of multiple predictive models based on NT-proBNP and adropin in assessing the risk of the transition from adverse cardiac remodeling to HFpEF. Nevertheless, further studies are needed to investigate the mechanistic, diagnostic, and prognostic roles of adropin levels in the transition from adverse cardiac remodeling to HFpEF.

However, there are some unresolved questions related to the relationship between adropin levels and their predictive ability in relation to the transition from adverse cardiac remodeling to HFpEF in individuals with T2DM. Although the obtained models had close discriminatory abilities, as evidenced by the multivariate analysis as well as the comparison of their AUCs, they were all based on low levels of adropin as the main discriminant. Moreover, even in the group of patients with elevated NT-proBNP, a reduced level of adropin allowed reliable reclassification into the group at risk of asymptomatic HFpEF occurrence. All these findings, in our opinion, confirm the fact that a reduced concentration of circulating adropin, regardless of the NT-proBNP concentration, is a promising predictor of asymptomatic HFpEF. Perhaps it is the comorbidity signature that determines the adropin levels in patients with asymptomatic adverse remodeling with normal or near-normal NT-proBNP levels. Indeed, Lian et al. (2011) [47] reported that BNP and BMI had independent impacts on the plasma adropin level and that the level of adropin increased in connection with the severity of chronic symptomatic HF. We did not notice that changes in the adropin level were mediated by NT-proBNP and BMI. On the contrary, among asymptomatic individuals with T2DM and HFpEF, low adropin levels were positively associated with the functional status of the myocardium (GLS) and negatively associated with the LAVI, BMI, and glucose homeostasis parameters. These findings are supported by other studies that have recruited high-risk HFpEF patients with comorbid metabolic conditions such as metabolic syndrome, obesity, and T2DM [21,22,23,24,29]. Thus, these findings suggest that adropin could be a promising predictive biomarker in patients with T2DM.

It is likely that adropin has a protective effect against diabetes-associated conditions such as angiopathy, multifocal atherosclerosis acceleration, obesity-induced nephropathy, and endothelial dysfunction, which independently contribute to the development and progression of HFpEF [48]. In addition, the relationship between adropin production and the excretory accumulation of perivascular and pericardial adipose tissue is not fully understood [49]. All this leaves considerable scope for future research in this direction.

Another important aspect for further consideration is the beneficial effects that some antidiabetic agents, such as dipeptidyl peptidase-4 inhibitors, SGLT2 inhibitors, and glucagon-like peptide-1 receptor agonists, have on glucose metabolism by increasing serum adropin levels [29,44,50,51,52]. They may be associated with cardioprotection and improvements in clinical status and outcomes in T2DM [52]. In our study, we did not find significant correlations between adropin and circulating inflammatory biomarkers, such as hs-CRT and TNF-alpha, whereas in other studies that mainly involved patients with non-alcoholic fatty liver disease, circulating adropin was inversely associated with inflammation and oxidative stress. On the contrary, we enrolled T2DM patients with well-controlled fasting glucose (HbAc1 < 6.9%) based on optimal antidiabetic therapy. However, patients treated with SGLT2 inhibition had a lower risk of developing HFpEF than those that were not [53,54]. As the evidence is weaker when it comes to the patient population with asymptomatic HFpEF and an eGFR < 60 mL/min/1.73 m^2^, there is a need to discover an effective tool to predict positive cardiac function responses when administering SGLT2 inhibitors to individuals with symptomatic HF. It should be noted that up to 50% of T2DM patients do not respond adequately to SGLT2 inhibitors in terms of organ protection [55,56]. Finally, a large clinical trial is needed to understand whether the transition from adverse cardiac remodeling to asymptomatic HFpEF is a target for treatment with SGLT2is in the T2DM population as a whole, regardless of atherosclerotic cardiovascular disease status.

## 5. Study Limitations

The limitations of this study mainly concern the lack of assessment of patients’ metabolic and nutritional statuses and the inclusion of T2DM patients with optimal glycemic control. However, we believe that these limitations will not affect the interpretation of the results.

## 6. Conclusions

In this study, we found that low levels of adropin (<3.5 ng/mL) in individuals with T2DM and known adverse cardiac remodeling significantly increased the predictive ability of NT-proBNP for asymptomatic HFpEF in the presence of near-normal levels of NT-proBNP (<125 pmol/L). This finding offers a new perspective for predicting the transition from adverse cardiac remodeling to HFpEF in diabetic patients, independent of the circulating levels of natriuretic peptides.

## Figures and Tables

**Table 1:** Basic characteristics of the patients involved in this study.

Variables	Entire Group (*n* = 561)	Low-NT-proBNP Group (*n* = 162)	Elevated-NT-proBNP Group (*n* = 399)	*p* Value
Age (years)	58 (47–70)	62 (52–68)	56 (44–69)	0.044
Male/female (*n* (%))	291 (51.7)/270 (48.2)	91 (56.2)/71 (43.8)	200 (50.1)/45 (49.8)	0.688
Diabetes duration (years)	11.20± 6.75	10.40± 6.80	11.90± 6.33	0.722
BMI (kg/m^2^)	26.90 ± 5.20	26.40 ± 2.70	27.10 ± 3.10	0.440
Waist circumference (cm)	96.60 ± 3.60	99.20 ± 3.70	91.10 ± 3.90	0.160
WHR (units)	0.88 ± 0.10	0.92 ± 0.09	0.84 ± 0.07	0.240
Dyslipidemia (*n* (%))	437 (77.9)	126 (77.8)	311 (77.0)	0.762
Hypertension (*n* (%))	415 (74.0)	124 (76.5)	291 (72.9)	0.430
Stable CAD (*n* (%))	173 (30.9)	44 (27.1)	129 (32.3)	0.020
Atrial fibrillation (*n* (%))	95 (16.9)	21 (12.9)	83 (20.8)	0.012
Smoking (*n* (%))	230 (41.0)	61 (37.7)	169 (42.4)	0.050
Abdominal obesity (*n* (%))	218 (38.9)	62 (38.3)	156 (39.1)	0.780
LVH (*n* (%))	509 (90.7)	141 (87.0)	368 (92.2)	0.050
CKD stages 1–3 (*n* (%))	151 (29.6)	43 (26.5)	108 (27.1)	0.344
Systolic BP (mm Hg)	137 ± 10	138± 9	136 ± 7	0.780
Diastolic BP (mm Hg)	84 ± 9	86 ± 7	81 ± 8	0.650
LVEDV (mL)	153 (146–160)	155 (139–161)	156 (143–164)	0.460
LVESV (mL)	71 (61–80)	73 (65–86)	69 (62–76)	0.058
LVEF (%)	54 (51–57)	53 (51–55)	56 (51–59)	0.054
LVMMI (g/m^2^)	139 ± 14	145 ± 11	132 ± 14	0.071
LAVI (mL/m^2^)	42 (35–50)	41 (36–48)	43 (38–49)	0.064
E/e′ (units)	16 ± 5	15 ± 4	18 ± 5	0.448
GLS (%)	−14.8 (−12.7; −15.7)	−15.3 (−12.4; −16.1)	−13.5 (−10.2; −15.7)	0.152
eGFR (mL/min/1.73 m^2^)	79 ± 17	68 ± 15	88 ± 11	0.046
HOMA-IR (units)	7.12 ± 2.7	7.07± 2.5	7.30 ± 2.6	0.740
Fasting glucose (mmol/L)	6.42 ± 1.9	6.30 ± 1.6	6.52 ± 1.7	0.812
HbA1c (%)	6.45 ± 0.18	6.25 ± 0.13	6.50 ± 0.19	0.565
Creatinine (µmol/L)	100.9 ± 20.1	94.1 ± 21.7	115.2 ± 30.1	0.640
SUA (mcmol/L)	321 ± 115	302 ± 75	385 ± 925	0.042
Total cholesterol (mmol/L)	5.98 ± 1.22	5.96 ± 1.20	6.02 ± 1.25	0.676
HDL-C (mmol/L)	0.96 ± 0.20	0.99 ± 0.18	0.93 ± 0.20	0.548
LDL-C (mmol/L)	3.31± 0.24	3.26 ± 0.21	3.35± 0.24	0.490
Triglycerides (mmol/L)	1.38 ± 0.22	1.35 ± 0.18	1.41 ± 0.24	0.480
hs-CRP (mg/L)	4.39 (1.98–6.80)	4.02 (2.10–6.22)	4.90 (2.25–7.22)	0.060
TNF-alpha (pg/mL)	2.70 (1.90–3.62)	2.42 (1.69–3.15)	2.98 (1.85–3.90)	0.056
NT-proBNP (pmol/mL)	147 (65–231)	87 (57–113)	188 (143–227)	0.001
Adropin (ng/mL)	3.50 (2.05–4.67)	4.20 (3.70–4.80)	2.40 (1.93–2.82)	0.001
ACE inhibitors (*n* (%))	221 (39.4)	48 (29.6)	173 (43.4)	0.042
Angiotensin-II receptor blockers (*n* (%))	207 (36.8)	49 (30.2)	158 (39.6)	0.050
Beta-blockers (*n* (%))	179 (31.9)	51 (31.4)	128 (32.1)	0.782
Ivabradine (*n* (%))	123 (21.9)	35 (21.6)	88 (22.1)	0.862
Calcium channel blockers (*n* (%))	161 (28.7)	45 (27.7)	116 (29.1)	0.438
Thiazide-like diuretics (*n* (%))	168 (29.9)	42 (25.9)	126 (31.6)	0.124
Antiplatelet agents (*n* (%))	173 (30.9)	64 (39.5)	109 (27.3)	0.020
Anticoagulants (*n* (%))	95 (16.9)	21 (12.9)	83 (20.8)	0.012
Metformin (*n* (%))	447 (79.8)	129 (79.6)	318 (79.7)	0.878
DPP4 inhibitors (*n* (%))	81 (14.4)	25 (15.4)	56 (14.0)	0.060
GLP-1 receptor agonists (*n* (%))	103 (18.4)	30 (18.5)	73 (18.3)	0.886
SGLT2 inhibitors (*n* (%))	492 (87.7)	129 (79.6)	363 (91.0)	0.048
Statins (*n* (%))	516 (91.2)	144 (88.9)	372 (93.2)	0.070

Notes: Variables are given as Ms ± SDs or Mes (25–75% IQRs). The Chi-square test was used to compare categorical variables. The Mann–Whitney U test, Kruskal–Wallis test, and Chi-square test were used to compare continuous variables between cohorts. LVH was detected when LVMI ≥ 95 g/m^2^ in women or ≥115 g/m^2^ in men. Abbreviations: BMI, body mass index; CAD, coronary artery disease; CKD, chronic kidney disease; DPP-4, dipeptidyl peptidase-4; eGFR, estimated glomerular filtration rate; E/e′, early diastolic blood filling to longitudinal strain ratio; GLS, global longitudinal strain; GLP-1, glucagon-like peptide-1; HbA1c, glycated hemoglobin; HDL-C, high-density lipoprotein cholesterol; HOMA-IR, Homeostatic Assessment Model of Insulin Resistance; hs-CRP, high-sensitivity C-reactive protein; LAVI, left atrial volume index; LDL-C, low-density lipoprotein cholesterol; LVH, left ventricular hypertrophy; LVEDV, left ventricular end-diastolic volume; LVESV, left ventricular end-systolic volume; LVEF, left ventricular ejection fraction; LVMMI, left ventricle myocardial mass index; NT-proBNP, N-terminal natriuretic pro-peptide; SGLT2, sodium–glucose cotransporter-2; SUA, serum uric acid; TNF-alpha, tumor necrosis factor-alpha; UACR, urinary albumin/creatinine ratio; WHR, waist-to-hip ratio.

**Table 2:** Predictors of HFpEF: the results of univariate and multivariate logistic regressions.

Variables		Dependent Variable: HFpEF						
	Univariate Cox Regression				Multivariate Cox Regression			
	HR	95% CI	*p*-Value	C-Index	HR	95% CI	*p*-Value	C-Index
Low NT-proBNP vs. elevated NT-proBNP	0.93	0.81–1.05	0.644	0.33	-			
Low adropin vs. elevated adropin	1.09	1.04–1.13	0.001	0.48	1.07	1.03–1.13	0.001	0.52
Low NT-proBNP + low adropin vs. elevated NT-proBNP	1.12	1.04–2.10	0.001	0.65	1.10	1.02–2.03	0.001	0.70
Low NT-proBNP + elevated adropin vs. elevated NT-proBNP	0.98	0.91–1.10	0.650	0.22	-			
Elevated NT-proBNP + low adropin	1.17	1.02–1.33	0.001	0.67	1.14	1.01–1.29	0.001	0.63
Elevated NT-proBNP + elevated adropin	1.05	1.00–1.12	0.224	0.46	-			
AF vs. non-AF	1.05	1.01–1.08	0.044	0.43	1.05	1.00–1.10	0.064	0.41
LVH vs. non-LVH	1.04	1.01–1.09	0.042	0.39	1.02	1.00–1.05	0.166	0.30
Administration of SGLT2i	0.91	0.86–0.98	0.044	0.34	0.94	0.88–0.96	0.041	0.38

Abbreviations: AF, atrial fibrillation; HR, hazard ratio; CI, confidence interval; LVH, left ventricular hypertrophy; SGLT2i, sodium–glucose cotransporter-2 inhibitor; NT-proBNP, N-terminal natriuretic pro-peptide.

**Table 3:** Comparison of predictive models for HFpEF.

Predictive Models	Dependent Variable: HFpEF					
	AUC		NRI		IDI	
	M (95% CI)	*p* Value	M (95% CI)	*p* Value	M (95% CI)	*p* Value
Model 1 (low adropin)	0.802 (0.773–0.857)	-	Reference	-	Reference	-
Model 2 (low NT-proBNP + low adropin)	0.783 (0.722–0.853)	0.122	0.28 (0.19–0.35)	0.213	0.23 (0.14–0.37)	0.426
Model 3 (elevated NT-proBNP + low adropin)	0.815 (0.801–0.833)	0.050	0.38 (0.22–0.51)	0.044	0.29 (0.17–0.41)	0.042
Model 4 (administration of SGLT2 inhibitors)	0.791 (0.760–0.862)	0.444	0.14 (0.10–0.19)	0.668	0.23 (0.19–0.29)	0.668

Abbreviations: AUC, area under curve; CI, confidence interval; NT-proBNP, N-terminal brain natriuretic pro-peptide; HF, heart failure; IDI, integrated discrimination indices; NRI, net reclassification improvement. Note: *p* value indicates a significant difference compared to Model 1.

## Data Availability

The data presented in this study are available on request from the corresponding author due to privacy restrictions.
